# Immune Checkpoint Inhibitor-Induced Miller Fisher Syndrome: A Case of Relapsing Symptoms Requiring a Slow Corticosteroid Taper

**DOI:** 10.7759/cureus.96247

**Published:** 2025-11-06

**Authors:** Miles Chapman, Muhammad A Khan, Rebecca Broad, Sreedharan Harikrishnan, Kaung Kyaw

**Affiliations:** 1 Neurology, East Kent Hospitals University NHS Foundation Trust, Canterbury, GBR

**Keywords:** avelumab, drug-induced neurotoxicity, guillain-barré syndrome, immune checkpoint inhibitor-induced mfs, immune checkpoint inhibitors, immune-related adverse events (iraes), merkel cell carcinoma (mcc), miller fisher syndrome, neurological irae, pd-l1 inhibitor

## Abstract

Immune checkpoint inhibitors (ICIs) have transformed oncology and are used across multiple malignancies, including Merkel cell carcinoma. However, ICIs have been increasingly recognized to trigger neurological immune-related adverse events. Miller Fisher syndrome is a rare variant of Guillain-Barré syndrome characterized by ophthalmoplegia, ataxia, and areflexia. We report the first fully described case of avelumab (a programmed death-ligand 1 (PD-L1) inhibitor)-associated MFS, highlighting its rarity and relapsing course. A 76-year-old man with metastatic Merkel cell carcinoma developed diplopia, ptosis, and ataxia shortly after two doses of avelumab. On examination, he had the classic clinical triad of ophthalmoplegia, ataxia, and areflexia. Cerebrospinal fluid analysis revealed albuminocytologic dissociation, anti-GQ1b antibodies were negative, and investigations for differential diagnoses such as myasthenia and paraneoplastic antibodies were negative. Nerve conduction studies demonstrated only mild sensory abnormalities without demyelination. He made substantial improvements with high-dose oral corticosteroids and plasma exchange but relapsed twice after steroid withdrawal (once after four months, and again after 14 months), requiring a slow taper of over one year. He has remained symptom-free on low-dose maintenance corticosteroids after two years of follow-up, and his Merkel cell carcinoma remains in complete remission after permanently discontinuing his immunotherapy and starting radiotherapy. Similar MFS presentations have been described with programmed cell death protein 1 inhibitors (such as pembrolizumab) but not previously with PD-L1 blockers. This case expands the spectrum of ICI-associated neuropathies, emphasizing that MFS due to PD-L1 inhibition may occur despite negative antibodies and subtle neurophysiological findings. Early immunosuppression, cautious corticosteroid tapering, and close multidisciplinary collaboration are essential for achieving sustained neurological and oncologic recovery.

## Introduction

Immune checkpoint inhibitors (ICIs), including programmed death-ligand 1 (PD-L1) inhibitors such as avelumab, have transformed the treatment of cancers, including Merkel cell carcinoma (MCC), urothelial carcinoma, and renal cell carcinoma. While they improve survival, ICIs can cause immune-related adverse events (irAEs) affecting multiple organ systems, including severe neurological complications such as myasthenia gravis, encephalitis, and Guillain-Barré syndrome (GBS) variants [1,2]. Neurological irAEs are uncommon, occurring in approximately 1-5% of patients, but can be serious and require prompt recognition and multidisciplinary management [1,2].

Miller Fisher syndrome (MFS) is a rare GBS variant defined by the triad of ophthalmoplegia, ataxia, and areflexia, and is classically associated with anti-GQ1b antibodies [3]. It is usually a post-infectious, self-limiting condition, although recurrence has been described in a minority of cases [3]. MFS lies within the broader GBS spectrum, sharing immune-mediated mechanisms that target peripheral nerves but differing in its cranial and proprioceptive predominance. ICI-associated MFS is exceptionally uncommon, with only a small number of cases described in the literature. Baird-Gunning et al. (2018) reported a demyelinating, anti-GQ1b-negative case after ipilimumab and nivolumab, Green et al. (2019) described an anti-GQ1b-negative case following pembrolizumab, and McNeill et al. (2019) detailed an anti-GQ1b-negative Fisher variant with initially normal but later axonal neurophysiology after nivolumab [4-6]. Taken together, published cases have mostly been seronegative and have shown variable neurophysiological findings, with many responding to corticosteroids, which are not typically effective in classical, post-infectious MFS. This suggests that ICI-related MFS may differ in both presentation and treatment from classical MFS.

Although MFS is listed as a rare adverse reaction to avelumab in its Summary of Product Characteristics [7], no fully described clinical case of avelumab-induced MFS has been published to date. The pathophysiological link between avelumab and MFS remains incompletely defined but likely involves loss of peripheral tolerance and T-cell-mediated autoimmunity triggered by PD-L1 blockade [1,8-10]. PD-L1 normally dampens immune activation through interaction with programmed cell death protein 1 (PD-1) on T cells; its inhibition enhances cytotoxic responses that may extend aberrantly to neural antigens [1,8-10]. We present the first such case, characterized by anti-ganglioside antibody negativity, subtle neurophysiological findings, and relapse after corticosteroid withdrawal, highlighting that prompt recognition of rare neurological irAEs is critical to guide diagnosis, management, and future research.

## Case presentation

**Figure 1:**
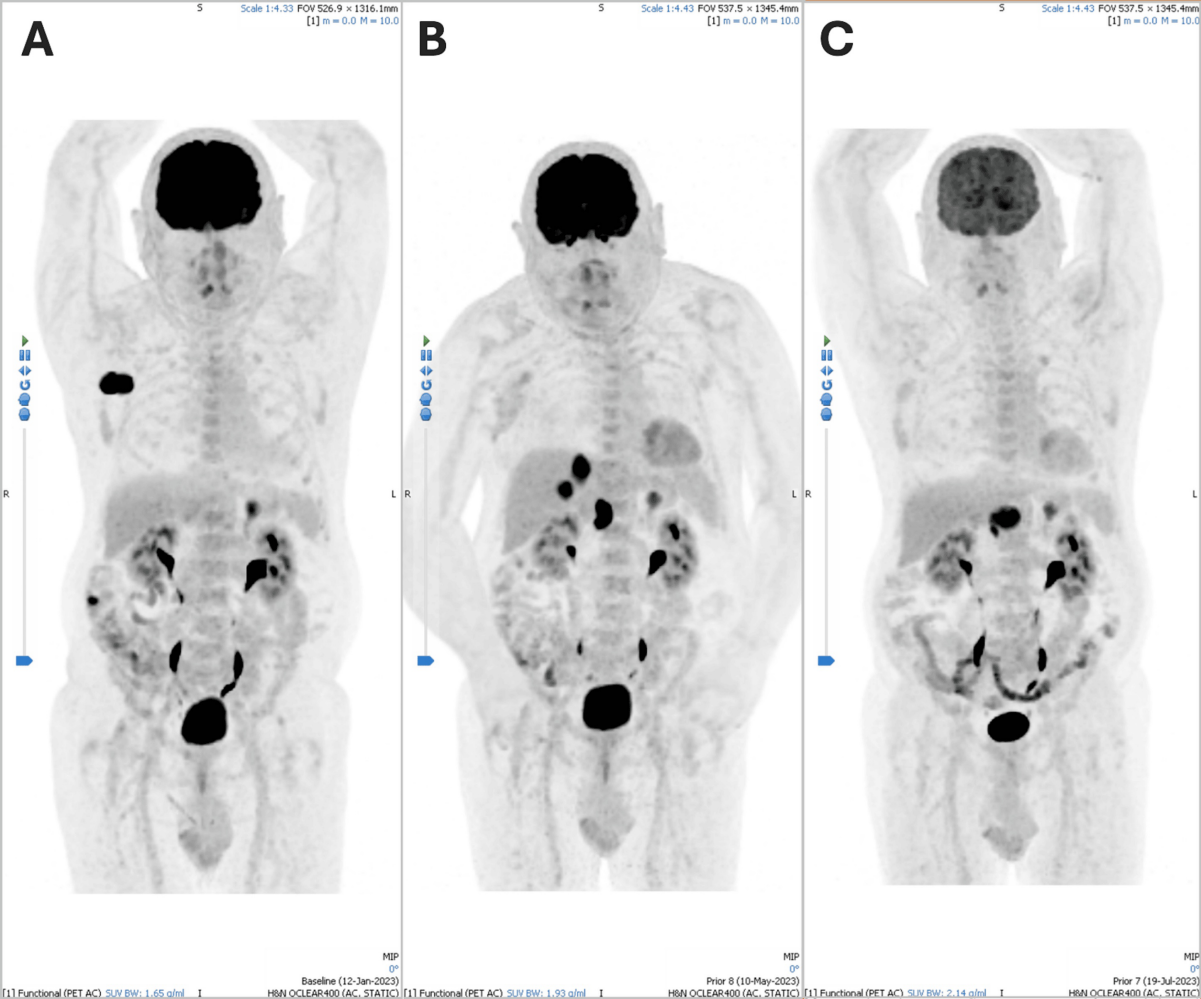
Positron emission tomography-computed tomography scan showing how the patient’s Merkel cell carcinoma was in remission at the time of his neurological symptoms. (A) The patient’s Merkel cell carcinoma (seen on his anterior right chest) at the time of his cancer diagnosis. (B) Excision of the chest lesion but metastases elsewhere, including the liver, shortly before initiating avelumab. (C) Image obtained on admission with his neurological symptoms, showing some remission of his Merkel cell carcinoma with improvement in his liver metastases after two doses of avelumab.

A 76-year-old man was diagnosed with high-grade neuroendocrine metastatic MCC. He had undergone excision of a cutaneous lesion, and a subsequent staging positron emission tomography-computed tomography (PET-CT) (see Figure 1, image B) showed nodal, hepatic, and adrenal metastases. He had no prior neurological disease, diabetes, or vitamin deficiencies, and was not taking any other medications known to cause neuropathy or demyelination, such as amiodarone. There was no history of recent infection, vaccination, or other immune stimulation before his symptoms.

He received two doses of first-line avelumab 800 mg, as an intravenous infusion, as per product recommendations two weeks apart [7]. He began experiencing double vision the day after his second infusion (see Table 1). Over the next two weeks, this progressed to ptosis and then subsequently lower back pain and unsteadiness. His symptoms continued to deteriorate to the point where he was struggling to walk, leading to his hospital admission one month after his first dose of avelumab.

**Table 1:** Timeline of the case.

Date/Interval	Key events and symptoms	Interventions	Outcome
Day 0 (May 2023)	First avelumab infusion	—	—
Day 14	Second avelumab infusion	—	—
Day 15	Diplopia begins	—	Symptom onset
Day 30 (June 2023)	Ptosis, ataxia, severe gait instability → hospital admission	Lumbar puncture (albuminocytologic dissociation); prednisolone 60 mg/day started; plasma exchange ×5 cycles	Marked improvement by the third cycle, mild diplopia persists
Week 6 (July 2023)	Discharge from hospital	Steroid taper: 60 mg × 1 week, then ↓5 mg/week until cessation	Symptom resolution by the end of July 2023
Week 18 (October 2023)	Relapse: diplopia, ptosis, ataxia	Prednisolone restarted 20 mg/day → escalated to 30 mg/day	Full resolution of symptoms by January 2024
July 2024	Miller Fisher syndrome relapse after steroid withdrawal, but complete cancer remission	Prednisolone restarted 15 mg/day, tapered slowly (↓5 mg/month)	Symptoms resolved by November 2024
September 2025	Remains symptom-free	Maintenance prednisolone 5 mg/day, tapering by 1 mg every two months	No relapse

On examination, the patient exhibited complete left ptosis, complex ophthalmoplegia, and impaired horizontal gaze. He had impaired proprioception in both upper and lower limbs, an ataxic gait, and the Romberg sign was positive. Areflexia was noted in all four limbs. Muscle power on admission was approximately Medical Research Council (MRC) grade 4+/5 in the upper and lower limbs, consistent with mild weakness, but improved to full strength by discharge. The combination of ophthalmoplegia, ataxia, and areflexia fulfilled the clinical criteria for MFS.

A lumbar puncture revealed elevated protein (2.08 g/L) with a normal white blood cell count, consistent with albuminocytologic dissociation (see Table 2). Anti-ganglioside antibodies (including anti-GQ1b) were negative, as were markers for myasthenia gravis (AChR, MuSK) and paraneoplastic syndromes (Hu, Ri, Yo, Ma2, CRMP5, Titin, Sox1, Zic4, Tr, Recoverin, VGCC antibodies) that formed part of our differential (see Table 2). Extensive infectious screening, including *Campylobacter jejuni*, cytomegalovirus, Epstein-Barr virus, and human immunodeficiency serologies, was negative. MRI of the brain and spine was unremarkable, whereas PET-CT demonstrated a mixed treatment response, including resolution of some hepatic lesions (see Figure 1). These findings effectively excluded other potential explanations, such as Bickerstaff brainstem encephalitis (absence of encephalopathy or hyperreflexia), paraneoplastic brainstem disease (negative antibody panel), and myasthenia gravis (negative AChR and MuSK antibodies with preserved compound muscle action potentials).

**Table 2:** Laboratory investigations.

Test	Patient’s result	Reference range	Units
Haemoglobin	13.2	13.0–17.5	g/dL
White blood cell count	6.2	4.0–11.0	×10⁹/L
Platelets	267	150–400	×10⁹/L
Sodium	138	135–145	mmol/L
Potassium	4.3	3.5–5.0	mmol/L
Creatinine	92	60–110	µmol/L
Alanine aminotransferase	32	<45	U/L
Aspartate aminotransferase	28	<40	U/L
C-reactive protein	3	<5	mg/L
Cerebrospinal fluid protein	2.08	0.15–0.45	g/L
Cerebrospinal fluid White cell count	2	<5	cells/µL
Anti-GQ1b antibody	Negative	Negative	—
Other ganglioside antibodies	Negative	Negative	—
AChR antibody	Negative	Negative	—
MuSK antibody	Negative	Negative	—
Paraneoplastic antibody panel (Hu, Ri, Yo, Ma2, CRMP5, Titin, Sox1, Zic4, Tr, Recoverin, VGCC)	Negative	Negative	—
Vitamin B12	520	200–900	pg/mL
HbA1c	5.4	<6.0	%

Nerve conduction studies (Table 3) showed preserved motor conduction velocities and amplitude, with attenuated but mostly recordable sensory responses in the sural and digital nerves. F-waves were intact, and there was no evidence of demyelination. Findings were interpreted by a consultant neurophysiologist as consistent with mild sensory neuropathy and non-diagnostic for classical MFS. Investigations for alternative causes of mild sensory neuropathy, including vitamin B12 and HbA1c, were normal. The absence of demyelination or axonal loss further supported an immune-mediated neuropathy distinct from classical post-infectious forms.

**Table 3:** Neurophysiology results on admission.

Nerve/Site	Type	Peak/Distal latency	Reference	Units	Amplitude	Reference	Units	Conduction velocity	Reference	Units
Sural (L) mid-calf → ankle	Sensory	3.45	<3.6	ms	2.7	>5	µV	—	>40	m/s
Sural (R)	Sensory	Not recordable	<3.6	ms	Not recordable	>5	µV	Not recordable	>40	m/s
Median (R digit II → wrist)	Sensory	3.69	<3.6	ms	4.1	>5	µV	42.7	>40	m/s
Ulnar (R digit V → wrist)	Sensory	2.38	<3.6	ms	1.91	>5	µV	51	>40	m/s
Peroneal (L) ankle → extensor digitorum brevis	Motor	3.45	<6.0	ms	1.87	>1.5	mV	50.2	>40	m/s
Ulnar (R) wrist → abductor digiti minimi	Motor	2.4	<4.0	ms	5.3	>3.0	mV	53.3	>40	m/s
Tibial (L) F-wave	Motor	14	<55	ms	—	—	—	—	—	—

The patient commenced oral prednisolone (60 mg daily) the day after admission, and then two days later started a five-cycle course of plasma exchange. Improvement in ophthalmoplegia and gait was noted by his third cycle. After two weeks in the hospital, his double vision had almost completely resolved, and he was discharged on a tapering corticosteroid regimen (reducing his prednisolone by 5 mg each week) with only mild imbalance and residual gaze-evoked diplopia.

Two months later, two weeks after completing his steroid course, he presented again with recurrent diplopia, left ptosis, and ataxia (see Table 1). Repeat nerve conduction studies again showed preserved motor conduction with mildly reduced sensory responses. He was promptly restarted on prednisolone (20 mg daily) and discharged. However, he required an increase to 30 mg daily a month later before his symptoms finally fully resolved.

Ten months after he first received avelumab, he completed his latest weaning regimen of prednisolone. He was then symptom-free for four months before relapsing once more (see Table 1). His prednisolone was restarted at 15 mg daily, then weaned by 5 mg monthly until a maintenance dose of 5 mg daily. He remained symptomatic for about four months before his symptoms resolved, and, to date, he remains asymptomatic. He has returned to independent ambulation and full self-care, with no residual visual or motor deficits reported, and his quality of life remains comparable to baseline. The prednisolone continues to be slowly weaned by 1 mg every two months.

Avelumab was permanently discontinued following the second dose, and whole-body radiotherapy 30 Gy was instead initiated two and a half months later for his MCC. He has been in complete metabolic remission from his cancer for over a year.

## Discussion

The pathophysiology of ICI-related MFS is incompletely understood but is likely driven by T-cell-mediated loss of peripheral immune tolerance, supported by its frequent seronegativity and responsiveness to corticosteroids [1,8]. Blockade of PD-L1 removes an inhibitory checkpoint that normally limits cytotoxic T-cell and helper T-cell activity against self-antigens. In the absence of this regulatory balance, autoreactive T-cells may target peripheral nerve components, particularly ganglioside-rich structures within cranial and sensory nerves. This results in the ophthalmoplegia, ataxia, and areflexia characteristic of MFS. Unlike post-infectious MFS, which is largely antibody-driven [3], these cases appear to represent a distinct, T-cell-dominant immune process with secondary, limited humoral activation. Importantly, ICI-related neurological toxicities can occur in clusters with other irAEs [2,9]; therefore, clinicians should actively screen for systemic involvement when such neurological presentations occur. In our patient, no concurrent endocrinological, dermatological, or gastrointestinal irAEs were detected, suggesting an isolated neurological manifestation.

Emerging data suggest mechanistic and clinical differences between PD-1 and PD-L1 inhibitors that may shape their toxicity profiles. Whereas PD-1 blockade simultaneously inhibits PD-1 interactions with both PD-L1 and PD-L2, producing a broader and less regulated T-cell response, PD-L1 inhibitors such as avelumab leave PD-L2 signaling intact, which may preserve partial immune homeostasis [10-14]. This mechanistic distinction may account for the generally lower frequency of systemic irAEs observed with PD-L1 inhibitors. Real-world studies appear to support this, reporting fewer treatment discontinuations and fewer irAEs overall [12,13]. In line with this, our patient developed an isolated neurological adverse reaction without systemic involvement.

Differential diagnosis in this case was broad and included Bickerstaff brainstem encephalitis, paraneoplastic brainstem syndromes, and myasthenia gravis. Bickerstaff was excluded by the absence of encephalopathy, hyperreflexia, or MRI abnormalities; myasthenia gravis was ruled out by negative AChR and MuSK antibodies and preserved neuromuscular transmission; and a paraneoplastic etiology was considered unlikely given negative antibody panels, stable tumor activity, and the close temporal relationship to avelumab exposure. The overall pattern of ophthalmoplegia, ataxia, areflexia, and albuminocytologic dissociation met the accepted diagnostic criteria for MFS [3].

Our case contributes to the small but growing literature on immunotherapy-associated MFS (see Table 4). The anti-GQ1b antibodies in our case were negative, consistent with the prior reports of Baird-Gunning et al. (2018), Green et al. (2019), and McNeill et al. (2019) [4-6]. However, neurophysiological findings varied among these studies. Baird-Gunning et al. reported a demyelinating polyneuropathy, and McNeill et al. described initially normal results that later evolved into reduced motor amplitudes suggestive of axonal involvement. In keeping with this variability, our patient’s studies were non-diagnostic, showing only subtle sensory changes. Taken together, these cases illustrate that serology and neurophysiology are not reliable discriminators in ICI-associated MFS. Instead, diagnosis rests on the characteristic clinical triad of ophthalmoplegia, ataxia, and areflexia, reinforced in our case by albuminocytologic dissociation in the cerebrospinal fluid. Importantly, to our knowledge, our case is the first report of relapsing ICI-associated MFS after corticosteroid withdrawal, highlighting the need for prolonged immunosuppression, cautious tapering, and close follow-up.

**Table 4:** Comparison of literature findings. ICI = immune checkpoint inhibitor; CTLA-4 = cytotoxic T-lymphocyte associated protein 4; PD-1 = programmed cell death protein 1; PD-L1 = programmed death-ligand 1; IVIG = intravenous immunoglobulin; PLEX = plasma exchange

Author	Age	ICI used	Mechanism of action	Anti-GQ1b	Neurophysiology	Treatment	Outcome	Relapse
Baird-Gunning et al., 2018 [4]	58 F	Ipilimumab + nivolumab	CTLA-4 + PD-1 blockade	Negative	Demyelinating polyneuropathy	IVIG + IV methylprednisolone; later PLEX; maintenance IVIG + prednisolone	Full recovery, persistent areflexia	No
Green et al., 2019 [5]	62 M	Pembrolizumab	PD-1 blockade	Negative	Not recorded	IVIG	Near complete recovery	No
McNeill et al., 2019 [6]	68 M	Nivolumab	PD-1 blockade	Negative	Initially normal → later ↓ motor amplitudes	IVIG (partial) → high-dose steroids	Substantial recovery, mild residual	No
Our case	76 M	Avelumab	PD-L1 blockade	Negative	Mild sensory changes only	Steroids + PLEX; prolonged taper	Multiple relapses → sustained remission	Yes

Management principles for ICI-associated MFS differ importantly from those of classical, post-infectious MFS, where corticosteroids are not recommended [15]. In contrast, our patient responded promptly to corticosteroids and plasma exchange, supporting early and aggressive immunosuppression in ICI-associated cases. Treatment followed the American Society of Clinical Oncology (ASCO) guidelines: withholding the ICI, initiating high-dose corticosteroids (prednisone 1-2 mg/kg/day equivalent), and escalating to intravenous immunoglobulin or plasma exchange for severe or refractory disease [9]. The decision to combine corticosteroids with plasma exchange as first-line therapy reflected these recommendations, the patient’s rapid clinical decline, and treatment availability. The escalation to intravenous methylprednisolone and intravenous immunoglobulins was considered but deferred due to early improvement, while rituximab was reserved for potential refractory relapse in line with ASCO guidance.

From an oncologic standpoint, avelumab was permanently discontinued in accordance with the Summary of Product Characteristics [7], European Medicines Agency guidance [10], and ASCO recommendations to cease immunotherapy after grade 3-4 neurological irAEs [9]. The patient was transitioned to whole-body radiotherapy, achieving durable cancer remission, and has remained under neurological and oncological follow-up for over two years. Long-term corticosteroid tapering was well tolerated, with no significant adverse effects. Immunotherapy rechallenge was avoided, given the severity of presentation, sustained remission with radiotherapy, and existing guidance discouraging reintroduction after severe neurological toxicity. This multidisciplinary approach exemplifies how coordinated care can ensure oncologic control while minimizing neurological risk.

Causality assessment using the Naranjo Adverse Drug Reaction Probability Scale yielded a score of 7, indicating a probable association between avelumab and MFS. The temporal relationship, lack of alternative causes, and relapse pattern after corticosteroid withdrawal further support this link. Overall, this case underscores the importance of early recognition, guideline-directed immunosuppression, and multidisciplinary coordination to optimize neurological and oncologic outcomes.

## Conclusions

This case demonstrates that ICI-associated MFS may be seronegative and present with subtle or even absent neurophysiological changes. Clinicians should prioritize recognition of the classical clinical triad of ophthalmoplegia, ataxia, and areflexia even when supportive investigations are inconclusive. Management differs from post-infectious MFS as corticosteroids are effective and, because relapse can occur after their withdrawal, careful tapering and close follow-up are essential. Finally, these neurological toxicities have significant implications for cancer therapy, underscoring the need for multidisciplinary collaboration to decide on permanent discontinuation of the ICI. Further research is required into the immunopathogenic mechanisms that underpin this adverse event, and to establish the optimal duration of immunosuppression in ICI-related neuropathies.
